# Esophageal perforation and septic shock after accidental coin ingestion in elderly person

**DOI:** 10.1002/ccr3.6703

**Published:** 2022-12-18

**Authors:** Yusei Ishizuka, Hironobu Nishiori, Yosuke Matsumura

**Affiliations:** ^1^ Department of Anesthesiology Chiba Emergency Medical Center Chiba Japan; ^2^ Department of Cardiovascular Surgery Chiba Emergency Medical Center Chiba Japan; ^3^ Department of Intensive Care Chiba Emergency Medical Center Chiba Japan

**Keywords:** coin, esophageal perforation, foreign body ingestion, mediastinitis, septic shock

## Abstract

An 88‐year‐old man ingested coins accidentally and developed mediastinitis and septic shock. Esophageal injuries by sharped‐shape foreign bodies are often reported, but esophageal perforation by round coins is rare. Even rounded‐shape foreign bodies that are unlikely to injure esophagus may lead to severe outcomes.

## Graphical abstract

A coin is a frequent cause of foreign body ingestion, which is usually expected spontaneous passing safely. Even round‐shaped foreign bodies may injure the esophagus developing mediastinitis and septic shock.

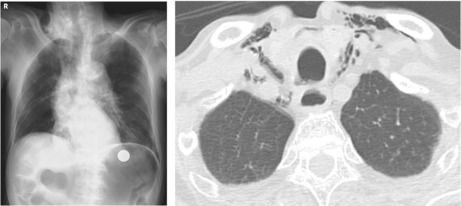

## 1 CASE

**FIGURE 1:**
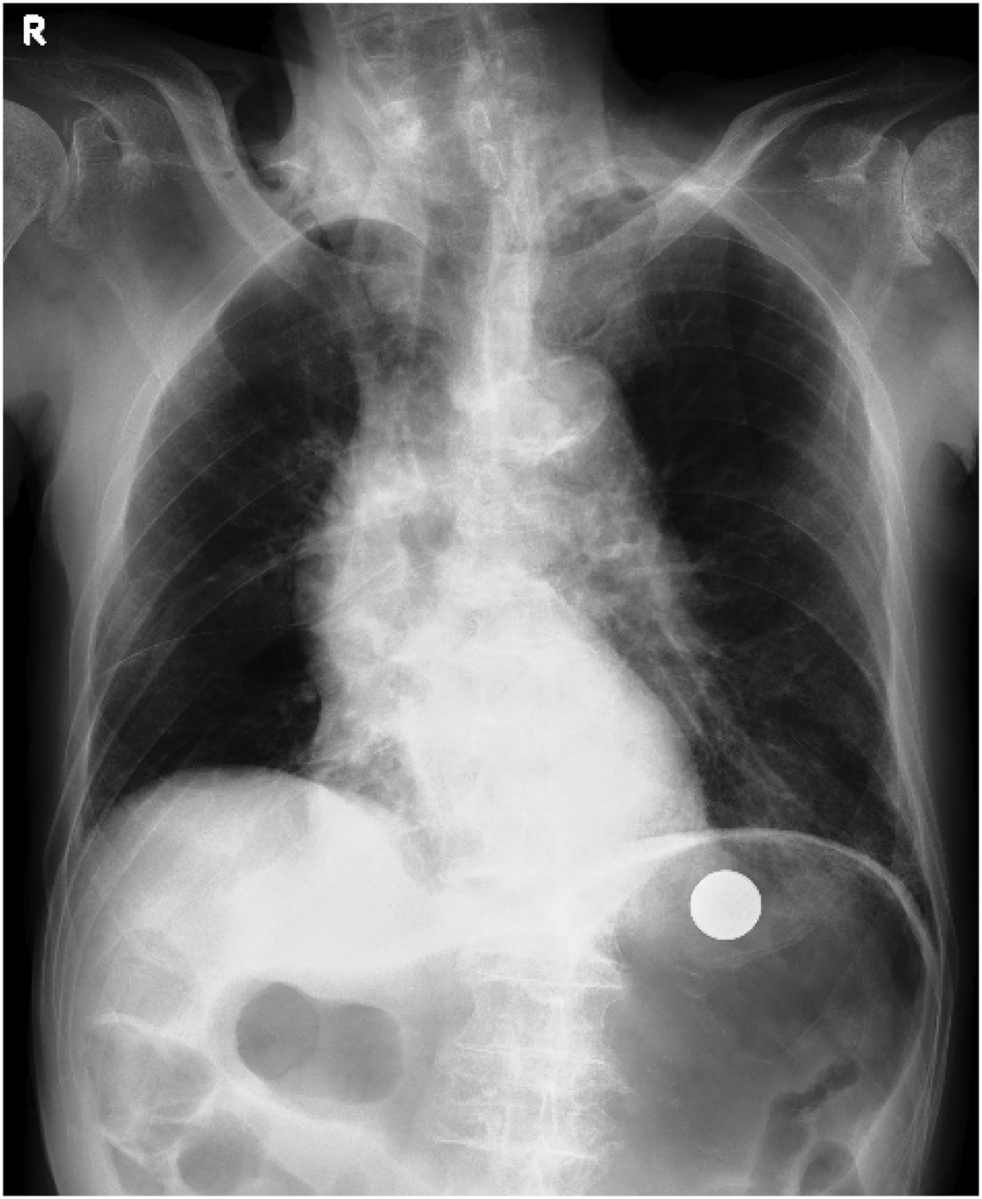
Radiolucent area was shown in the mediastinum and a clear metallic shadow was seen in the stomach.

**FIGURE 2:**
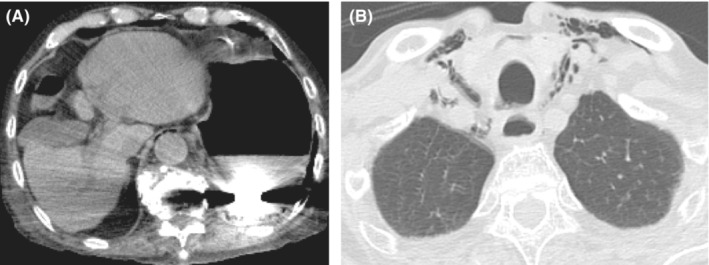
(A) Hyperintense foreign body was shown in the stomach. (B) Air was observed around the esophagus and in the anterior mediastinum.

**FIGURE 3:**
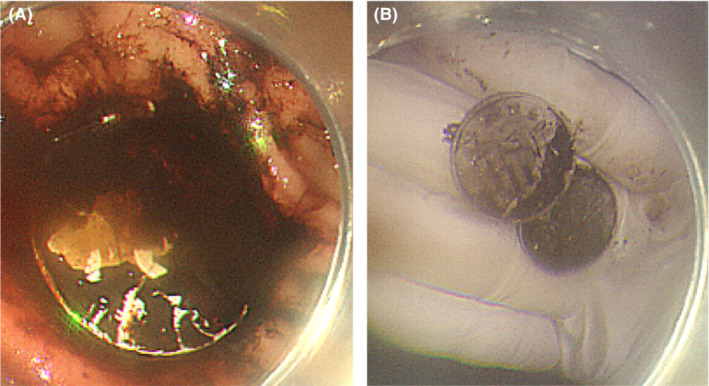
(A) Coins were embedded in the stomach body. (B) Two coins were removed endoscopically, although they appeared to be a single coin on X‐ray and CT.

An 88‐year‐old man was transferred by ambulance with hypotension and impaired consciousness. X‐ray showed a radiolucent area around the mediastinum and a coin in the stomach (Figure 1). Computed tomography imaging revealed mediastinal emphysema and a hyperintense gastric foreign body (Figure 2A,B). The patient was diagnosed with septic shock due to esophageal perforation by accidental ingestion of coins and subsequent mediastinitis. The patient was managed with antibiotics and esophageal drainage, and his physical condition showed improvement. On Day 25, the patient developed hemorrhagic gastric ulcer after prolonged fasting, and endoscopic clipping was performed in the pyloric region. Two coins (22.6 mm diameter) were removed from the gastric body region (Figure 3A,B). After the hemostasis and transfusion, he developed aspiration pneumonia and acute respiratory distress syndrome, then passed away on Day 34.

Coins are one of the most common esophageal foreign bodies in adults, and it is usually expected spontaneous passing safely. Esophageal perforation due to foreign bodies is rare at 0.91%.^1^ Also, ingestion of coins has been reported to cause otalgia.^2^ While esophageal injuries after sharp edges ingestion, including fish bones and press‐through‐packs, are often experienced, perforation after rounded‐shape coin ingestion is rarely reported.^3^ Even foreign bodies considered unlikely to injure the esophagus may cause perforation and lead to a critical course.

## AUTHOR CONTRIBUTIONS

**Yusei Ishizuka:** Writing – original draft; writing – review and editing. **Hironobu Nishiori:** Supervision; writing – review and editing. **Yousuke Matsumura:** Supervision; writing – review and editing.

## FUNDING INFORMATION

None.

## CONFLICT OF INTEREST

None declared.

## ETHICS STATEMENT

None.

## CONSENT

Written informed consent was obtained from the patient to publish this report in accordance with the journal's patient consent policy.

## Data Availability

None.
